# The Removal of CH_4_ and NO*_x_* from Marine LNG Engine Exhaust by NTP Combined with Catalyst: A Review

**DOI:** 10.3390/ma16144969

**Published:** 2023-07-12

**Authors:** Neng Zhu, Yu Hong, Yunkai Cai, Fei Dong, Jie Song

**Affiliations:** 1 School of Automotive and Transportation Engineering, Wuhan University of Science and Technology, Wuhan 430081, China; znqc@wust.edu.cn (N.Z.); hyhyjyjy2000@163.com (Y.H.); 2 School of Naval Architecture, Ocean and Energy Power Engineering, Wuhan University of Technology, Wuhan 430063, China; 3 Weichai Power Co., Ltd., Weifang 261061, China; songjie@weichai.com

**Keywords:** marine, LNG engines, CH_4_ emission, NO*_x_* emission, nonthermal plasma, catalyst

## Abstract

Compared to diesel, liquefied natural gas (LNG), often used as an alternative fuel for marine engines, comes with significant advantages in reducing emissions of particulate matter (PM), SO*_x_*, CO_2_, and other pollutants. Promoting the use of LNG is of great significance for achieving carbon peaking and neutrality worldwide, as well as improving the energy structure. However, compared to diesel engines, medium- and high-speed marine LNG engines may produce higher methane (CH_4_) emissions and also have nitrogen oxide (NO*_x_*) emission issues. For the removal of CH_4_ and NO*_x_* from the exhaust of marine LNG engines, the traditional technical route of combining a methane oxidation catalyst (MOC) and an HN_3_ selective catalytic reduction system (NH_3_-SCR) will face problems, such as low conversion efficiency and high operation cost. In view of this, the technology of non-thermal plasma (NTP) combined with CH_4_-SCR is proposed. However, the synergistic mechanism between NTP and catalysts is still unclear, which limits the optimization of an NTP-CH_4_-SCR system. This article summarizes the synergistic mechanism of NTP and catalysts in the integrated treatment process of CH_4_ and NO*_x_*, including experimental analysis and numerical simulation. And the relevant impact parameters (such as electrode diameter, electrode shape, electrode material, and barrier material, etc.) of NTP reactor energy optimization are discussed. The work of this paper is of great significance for guiding the high-efficiency removal of CH_4_ and NO*_x_* for an NTP-CH_4_-SCR system.

## 1. Introduction

Ship transportation undertakes over 90% of global transportation tasks [1]. Marine engines using traditional petroleum or heavy oil as fuel can cause environmental damage and air pollution, such as nitrogen oxides (NO*_x_*), SO*_x_*, particulate matter (PM), etc. [2]. Choosing alternative fuels (such as methanol, bioethanol, natural gas, etc.) is an effective way to reduce the exhaust pollution of marine engines [2,3,4]. As an alternative fuel for marine engines, liquefied natural gas (LNG, mainly composed of CH_4_) has shown significant advantages in reducing emissions such as PM and SO*_x_* [5,6]. At the same time, LNG has a lower C/H ratio, which can reduce CO_2_ emissions by about 30% compared to diesel [7]. Promoting the use of LNG is of great significance for achieving carbon peak and carbon neutrality worldwide and is also important for improving the energy structure.

However, compared to diesel engines, medium- and high-speed marine LNG engines may produce higher methane (CH_4_) emissions and also have NO*_x_* emission issues [8,9]. CH_4_ is a kind of greenhouse gas, and its halocarbon global warming potential (HGWP) is about 28 times that of CO_2_ [10]. NO*_x_* is one of the important factors that cause harmful weather, such as photochemical smog, haze, and acid rain. Long-term inhalation of NO*_x_* can cause serious damage to the functions of human viscera and even threaten human life. The CH_4_ and NO*_x_* emissions from several types of high-speed marine LNG engines developed by Guangxi Yuchai Machinery Group Co., Ltd. (Guangxi, China) were tested and collated, as shown in Table 1.

In order to control the CH4 and NO*_x_* emissions from marine LNG engine exhaust, China has issued the “Limits and measurement methods for exhaust pollutants from marine engines (CHINA I, II)” [11], in which the CHINA II stage requires a CH_4_ emission limit of 1.0 to 2.0 g/kWh. IMO Tier III requires that the NO*_x_* emissions of medium- and high-speed marine engines (1000–2000 r/min) should be limited to 2.26–1.96 g/kWh. Moreover, the Euro VI imposes stricter requirements on CH_4_ and NO*_x_* emissions from heavy-duty natural gas engines [12], which means that there is a possibility that the emission standards for ship engines will become increasingly strict. Considering the increasingly stringent constraints on pollutant emissions in the future, it is necessary to deal with CH_4_ and NO*_x_* emissions from marine LNG engines.

Due to the lean combustion strategy, medium- and high-speed marine LNG engines may have a lower exhaust temperature. And the oxygen-enriched condition of marine-engine exhaust limits the use of three-way catalysis (TWC) [13]. For the removal of CH_4_ and NO*_x_* emissions from the exhaust gas of medium- and high-speed marine LNG engines, the traditional technical route of combining a methane oxidation catalyst (MOC) and an HN_3_ selective catalytic reduction system (NH_3_-SCR) will lead to the following problems: (1) A large number of noble metal catalysts, such as platinum, palladium, and rhodium, are used in MOC, resulting in high economic costs; and (2) The low exhaust temperature will lead to the low catalytic oxidation efficiency of CH_4_ [14], and this problem will be exacerbated under low load conditions. Moreover, MOC combined with the SCR system has a large mass and dimensions, which is difficult to install on ships with limited space. And the use of pure ammonia on marine vessels is hazardous and requires special attention.

Some studies have shown that CH_4_ in the exhaust of LNG engines can be used as a reducing agent to remove NO*_x_* from the exhaust gas. Therefore, selective catalytic reduction using CH_4_ as a reducing agent (CH_4_-SCR) has been studied as a desirable technology for the removal of NO*_x_* and CH_4_ simultaneously in marine LNG engine exhaust [15]. Compared to the MOC+NH_3_-SCR technology route, the CH_4_-SCR system has the significant advantages of low cost, a small footprint, and a simple system [16]. However, due to the high stability of CH_4_ molecules and the poor low-temperature activity of the CH_4_-SCR system, its application in the integrated treatment of CH_4_ and NO*_x_* from marine LNG engine exhaust is limited [17]. To improve the low-temperature activity of catalytic systems, non-thermal plasma (NTP) technology was introduced into the traditional catalytic field [18]. The electron temperature in non-thermal plasma is very high (in the order of 10^4^–10^5^ K); the temperature of heavy particles (ions and neutrals) is around 300–1000 K, and the whole system presents a low-temperature state, so it is also called cold plasma, or non-equilibrium plasma. The generation of NTP requires additional energy consumption, which is the key factor affecting the commercial application of the NTP+CH_4_-SCR system [19]. And optimizing system energy consumption and improving system conversion efficiency requires us to fully understand the synergistic mechanism between NTP and CH_4_-SCR catalysts.

## 2. The Introduction of NTP Technology in the CH_4_-SCR System

**Figure 1:**
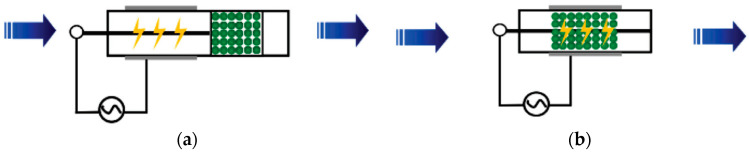
Two types of NTP synergistic catalytic reactors: (**a**) PPC Reactor and (**b**) IPC Reactor.

For reactions that require high activation energy, the average energy of high-energy electrons generated in NTP is 1 to 10 eV. The high-energy electrons can activate and dissociate gas molecules through inelastic collisions, making many reactions occur that usually require extremely harsh reaction conditions [20]. According to the arrangement of the plasma and catalyst, NTP synergistic catalytic reactors can be divided into two types: post-plasma catalyst (PPC) and in-plasma catalyst (IPC), as shown in Figure 1.

**Figure 2:**
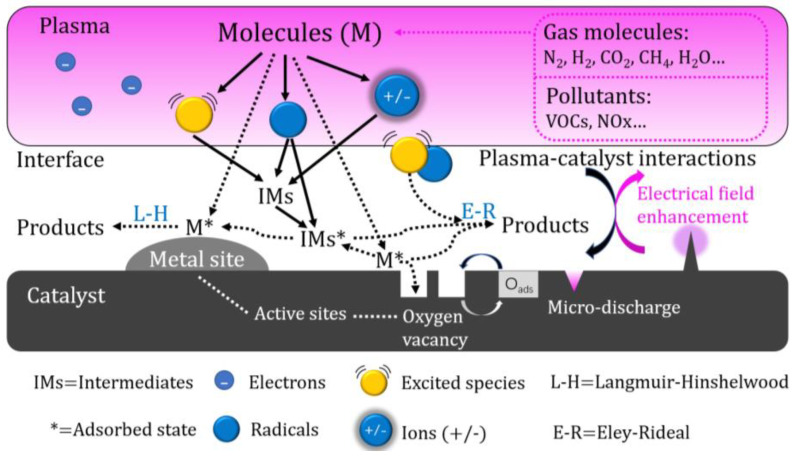
The Synergistic Mechanism of Plasma and a Catalyst in IPC Reactor.

Due to the simultaneous occurrence and interaction of the plasma and catalytic processes in the IPC reactor, the reaction process is more complex and efficient than the reaction in the PPC reactor [20]. In the IPC reactor, the catalyst is arranged in the plasma discharge space, and the pores inside the catalyst will generate plasma in the form of a micro discharge, improving the density of the plasma. The short-lived active species generated by a plasma discharge (such as excited molecules and radicals) can more effectively act on the catalyst and improve the treatment efficiency of the catalyst [21,22]. Figure 2 shows the interaction mechanism between plasma and a catalyst in the IPC reactor.

Previous research has demonstrated the significant effect of NTP in producing active species and reducing reaction temperature. CH_4_ oxidation and NO*_x_* reduction are complementary processes, and there are relevant studies on CH_4_-SCR systems aimed at NO*_x_* removal [23,24]. Therefore, adopting the synergetic method of NTP and catalysts (NTP-CH_4_-SCR) to achieve the integrated treatment of CH_4_ and NO*_x_* from the exhaust of marine LNG engines has become a research hotspot.

On the one hand, the introduction of NTP technology can reduce the reaction temperature of CH_4_ and NO*_x_* removal to overcome the low-temperature limitations of marine LNG engine exhaust. On the other hand, compared to MOC+NH_3_-SCR, NTP and catalyst synergism to treat CH_4_ and NO*_x_* using only one catalyst can reduce the use of noble metal catalysts, which is of great significance in improving the economy of exhaust after-treatment devices.

Therefore, using the method of synergic NTP and catalyst to achieve the simultaneous removal of CH_4_ and NO*_x_* from marine LNG engines is an effective way to overcome the low exhaust temperature and reduce the use of noble metal catalysts. However, in practical applications, the energy consumption of the NTP synergic catalyst system must be considered, which is related to the system economy. This requires careful consideration of the optimal design of NTP reactors and catalysts suitable for plasma environments [22]. Fully understanding the synergistic mechanism of NTP and catalysts is a prerequisite. At present, the synergistic mechanism between NTP and catalysts is still unclear, which limits the optimization and promotion of energy consumption of the NTP-CH_4_-SCR system.

## 3. Development Status of NTP Synergistic Catalyst for CH_4_ and NO*_x_* Treatment

This part summarizes the synergistic mechanism of NTP and catalysts in the integrated treatment of CH_4_ and NO*_x_* (including research based on experimental measurements and research based on simulation models) and discusses the relevant influencing parameters for energy consumption optimization of NTP reactors.

### 3.1. Research on the Synergistic Mechanism of NTP and Catalyst Based on Experiment

The complex synergistic mechanism of NTP-and-catalyst interactions limits the optimization and commercialization of NTP-CH_4_-SCR systems. Due to the different reaction mechanisms between NTP and catalysts, most conventional thermal catalysts are not suitable for the plasma environment (especially in IPC reactors). In addition, most studies focus on the performance of conversion efficiency, yield, or selectivity of plasma catalysis. Detailed characterization information on plasma and catalyst surface states is lacking. Moreover, plasma catalysis involves many coupling processes, such as fluid mechanics, heat transfer, surface reactions, plasma chemistry, and active species diffusion. This makes it difficult to establish a numerical simulation model, especially for more efficient IPC systems. And full understanding of the synergistic mechanism of NTP and catalyst interaction is limited. Therefore, it is particularly important to carry out systematic diagnosis and experiments on the NTP-CH_4_-SCR system.

In 2003, Chen et al. [25] experimentally studied the process of CH_4_ reduction of NO in a PPC reactor, which used γ-Al_2_O_3_ as a CH_4_-SCR catalyst. The experimental result showed that the removal efficiency of NO*_x_* by the PPC system was about 15% higher compared to that by the catalyst alone. And the synergistic mechanism of a plasma-assisted catalyst was proposed. In 2005, Niu et al. [18] also found a synergistic effect between NTP and CH_4_-SCR catalysts in a PPC reactor. In 2009, Li et al. [26] conducted research on the catalytic reduction of NO by CH_4_ in a PPC reactor. The experimental results show that the introduction of NTP could effectively improve the low-temperature activity of the system, and there was a synergistic effect between NTP and the catalyst. To analyze the impact of NTP on the catalyst, transmission electron microscopy (TEM), H_2_-temperature-programmed reduction (H_2_-TPR), X-ray diffraction (XRD), and X-ray photoelectron spectroscopy (XPS) were used to investigate the catalyst characteristics before and after the reactions.

**Figure 3:**
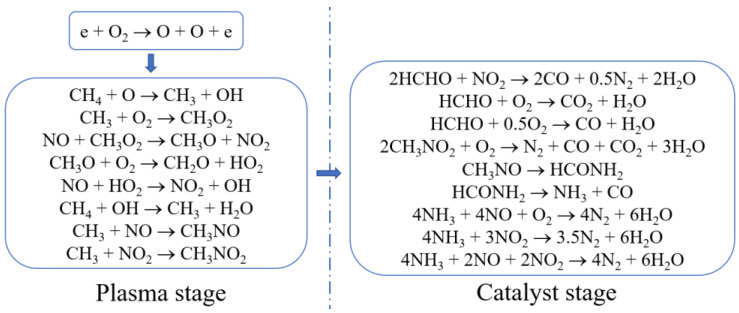
Mechanism of NO*_x_* reduction by CH_4_ in NTP-CH_4_-SCR system proposed by Yu et al. [27].

In order to diminish NO*_x_* emissions from stationary sources, Yu et al. [27] studied the mechanism of NO*_x_* reduction by CH_4_ in a PPC reactor and simulated exhaust composed of 0.8% NO, 0.4% CH_4_, 3% O_2_, and He (balance). The results of temperature-programmed surface reaction (TPSR) experiments show that the reactants were activated by NTP to generate active intermediates such as NO_2_, HCHO, CH_3_NO, and CH_3_NO_2_. These intermediates can further react on the catalyst to convert NO*_x_* to N_2_. Based on the experimental results, the following mechanism of NTP synergistic catalytic reaction was proposed (as shown in Figure 3).

In 2015, Pan et al. [28] studied the effect of NTP on the reduction of NO*_x_* by CH_4_ over In/H-BEA catalysts in a PPC reactor. The experimental results show that NTP could significantly improve the low-temperature activity of the catalyst and reduce the negative impact of H_2_O and SO_2_. It is speculated that the OH radicals generated by the decomposition of H_2_O in the NTP reactor promoted the conversion and condensation of SO_2_ to H_2_SO_4_ and H_2_SO_3_ and reduced the formation of sulfate in the catalyst. However, this may result in sulfide corrosion, which is dangerous at low temperatures. The synergistic mechanism of NTP still needs further research.

In 2015, Steel et al. [29] developed a DRIFTS-MS (diffuse reflectance infrared Fourier transform spectroscopy combined with mass spectrometry) system to study the synergistic mechanism of NTP and catalysts. DRIFTS-MS is used to measure the changes in species on the surface of silver-based catalysts during the NTP synergistic HC-SCR denitrification process. The research determined the importance of isocyanate species in the HC-SCR denitrification reaction, as well as the key role of water in the formation of N_2_. This demonstrates the potential of the DRIFTS-MS system in exploring the interaction mechanism between NTP and catalysts. In 2016, Rodrigues et al. [30] developed an IPC reactor suitable for the DRIFTS system. The decomposition process of isopropanol and toluene on the catalyst surface was observed by in situ detection.

In terms of CH_4_ catalytic reforming, Knoll et al. [31] studied the CH_4_ decomposition by partial oxidation reaction in a PPC reactor, which was made by combining the atmospheric pressure plasma jet (APPJ) with a Ni catalyst. Fourier-transform infrared spectroscopy analysis of diffuse reflectance of the gas phase products and a DRIFTS in situ analysis of the catalyst surface were used simultaneously. The experimental result indicated that the formation of CO and other carboxylate groups (bonded to the catalyst surface, IR spectral feature at 1590 cm^−1^) was related to NTP discharge. In 2022, this research team [32] investigated APPJ-assisted CH4 oxidation over a Ni catalyst in a PPC reactor. The catalyst surface was characterized by in situ DRIFTS. The result of different exposure conditions (with or without NTP) of the catalyst indicated that the reduction of the catalyst by the APPJ was likely the cause of the catalyst activation. In addition, when the plasma operating conditions were varied, a systematic change in the vibrational frequency of adsorbed CO on the catalyst was observed. This work provides insights into the interactions between plasma and catalysts, especially the modification of catalysts during plasma catalytic processes.

**Figure 4:**
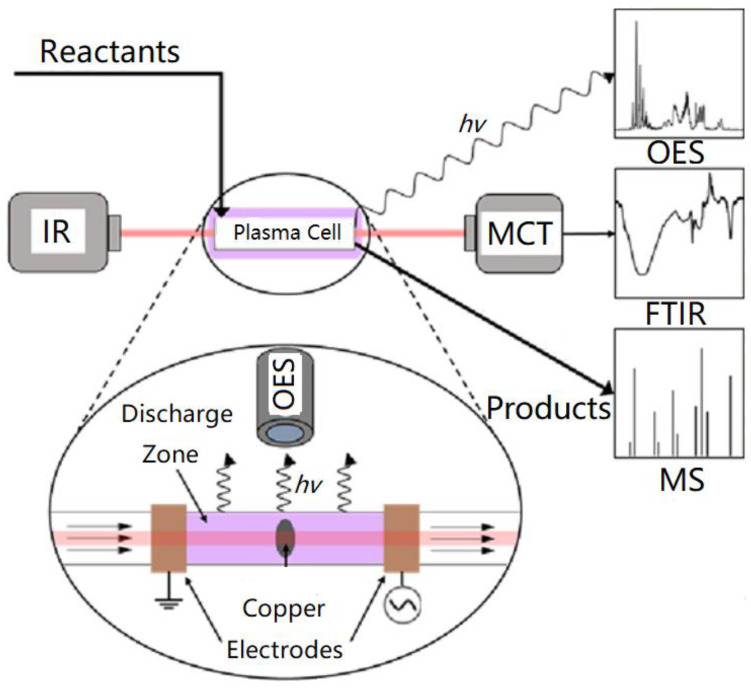
Schematic diagram of FTIR, OES, and MS system for IPC reactor.

Optical emission spectroscopy (OES) analysis is an important method for in situ detection of plasma characteristics. In 2017, Du et al. [33] measured the temperature distribution in the IPC reactor during the CH_4_/CO_2_ reforming process based on the OES of CO(B^1^Σ^+^→A^1^Π). In 2022, Clarke et al. [34] designed an IPC reactor capable of connecting with Fourier transform infrared spectroscopy (FTIR), OES, and mass spectra (MS), while characterizing the catalyst surface, plasma, and gas phase, as shown in Figure 4. Using this system, the evolution of NO*_x_* species bound to Pt/SiO_2_ surfaces under plasma action was detected. It confirmed its potential as an important tool for studying the interaction mechanism between NTP and catalyst.

Cai et al. [35,36] used the OES of NTP to measure the active species (O and OH radicals) generated in plasma and analyzed the impact mechanism of various gas components (O_2_, H_2_O, CO_2_, and C_3_H_6_) on the removal of NO and SO_2_. This research verified the important role of OES of NTP in studying the mechanism of plasma reactions. Li et al. [37] studied the effects of various gas components on plasma-assisted catalysts for the removal of CH_4_ and NO in a PPC reactor, and the synergistic mechanism between NTP and catalysts was analyzed through experiments.

In general, it is necessary to analyze the synergistic mechanism between NTP and catalysts based on the measured steady-state products and the intermediates adsorbed on the catalyst surface. However, it is difficult to fully analyze the role of short-lived, highly active species in the synergistic process between NTP and catalysts, only relying on the information obtained from experimental diagnostics.

### 3.2. Research on Synergistic Mechanism of NTP and Catalysts Based on Simulation Model

**Figure 5:**
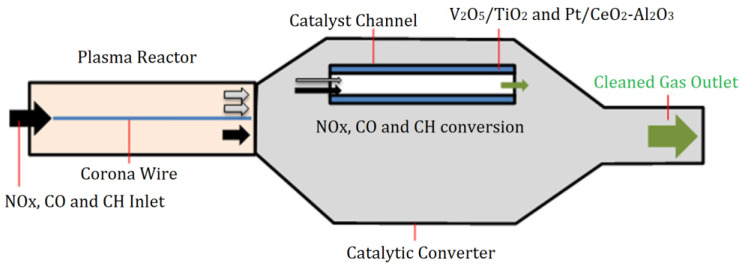
Schematic diagram of 2D model of PPC reactor.

The interaction between NTP and catalysts is very complex, including the impact of the catalyst on plasma discharge, active species generation, and the impact of activated species in plasma on the reaction path of the catalyst surface. In order to fully analyze the interaction mechanism between NTP and catalysts, it is necessary to establish a numerical simulation model. From 2016 to 2018, Annemie et al. [38,39] established a 2D fluid model to study the discharge process within the micron-scale catalyst hole in an IPC reactor based on COMSOL Multiphysics. The results show that plasma can be formed in the catalyst pores, which may interact with the catalyst surface and affect the plasma catalytic process. In 2019, in the field of HC and NO*_x_* treatment, Oskooei et al. [40] first applied COMSOL Multiphysics to establish a 2D fluid model (as shown in Figure 5) for a PPC reactor. Four electron collision reactions and six catalyst surface reactions (including four DOC reactions and two NH_3_-SCR reactions) were considered in this model. The effect of electric field intensity on the concentration of NO*_x_*, C_3_H_6_, and radical generation was calculated, and the synergistic effect of NTP and catalysts was preliminarily simulated. In the field of plasma-enhanced chemical vapor deposition (PECVD), Zhang and Gupta et al. [41,42] established a dynamic model for the growth of a single carbon nanofiber in the cathode layer of glow-discharge plasma. The chemical kinetics, heat transfer, and electric field distribution dynamics are considered in the model. Based on numerical models, the influence and mechanism of strong electric fields on the growth of nanofibers were studied. This improved the understanding of plasma-assisted nanofabrication and demonstrated the role of numerical models in studying the synergistic mechanism of plasma. Bai et al. [43] established a one-dimensional fluid model and conducted a numerical simulation of the CH_4_/CO_2_ plasma reaction under atmospheric pressure conditions. This model included 68 species and 276 reactions. And discharge current density, discharge gap voltage, dissipative power density, spatial average particle density, distribution of high-density species, and the reaction pathways of important species in dielectric barrier discharge (DBD) reactor were systematically discussed. This provides a valuable reference for exploring the mechanism of plasma-assisted catalyst reactions.

In 2020, Xiong et al. [44] built a 2D particle­in­cell/Monte Carlo collision model for an IPC reactor with five different packing-bead methods at atmospheric pressure in N_2_ gas. Based on this model, the relationship between discharge characteristics and dielectric beads was studied. And the effect of different packing-bead methods on the electron density, electric field, excitation rate, and ionization rate was discussed. This work is helpful for understanding the synergistic mechanism of plasma catalyses and improving the efficiency of plasma catalyses. In flue gas catalytic desulfurization, Ning et al. [45] used CuO-ZrSnO_4_ as a catalyst and investigated the effect of NTP on improving the low-temperature efficiency of the catalyst in a PPC reactor. Through density functional theory (DFT) calculation of the catalyst and numerical simulation of the NTP reactor based on COMSOL, the chemical kinetic reaction was analyzed. Further, in IPC and PPC reactors, Ning et al. [46] investigated the effects of plasma-catalysis coupling on sulfur-removal efficiency. The results showed that the removal efficiency of SO_2_ in PPCs was improved by 20–30% by NTP, while the removal efficiency of SO_2_ in IPC was higher than that in PPC. In order to understand the synergistic mechanism between NTP and the catalyst, the catalytic behavior of the catalyst during the thermal and NTP reactions was analyzed by X-ray diffraction, scanning electron microscope, and X-ray photoemission spectroscopy. The OES of NTP and the numerical model based on COMSOL were used to study the reactive species and their transformation processes in discharge space [46,47]. However, the separated numerical calculations of NTP and the catalyst do not adequately resolve the interaction mechanisms between them.

Fully understanding the highly complex interactions between plasma and catalytic materials is important to improving plasma energy efficiency and conversion efficiency. Cheng et al. [48,49] developed a new numerical method to investigate how ns pulse-driven plasma triggers the heterogeneous reactions on the surface of the Ni/γ-Al_2_O_3_ catalyst as well as the discharge form in an IPC reactor. The numerical model showed that the high electron power in the surface ionization wave region and the high ion power density in the catalyst surface sheath region provided enough energy to trigger the heterogeneous reaction.

**Figure 6:**
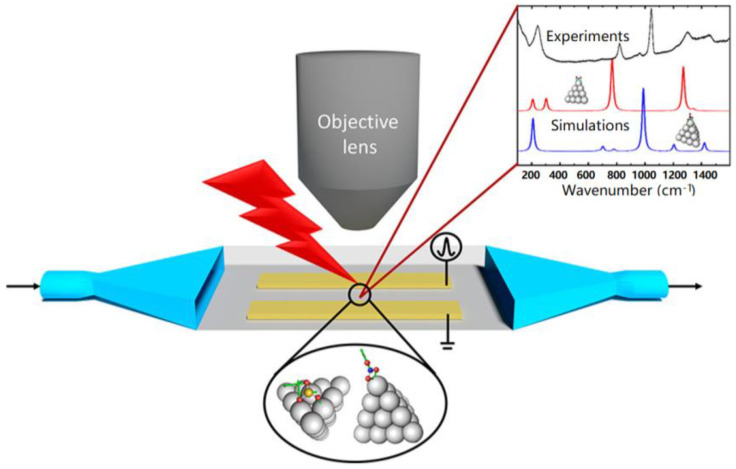
SERS Spectrometric Measurement of Key Reaction Intermediates on the Surface of Silver Nanoparticles.

In 2021, in the field of desulfurization and denitrification from marine diesel engine exhaust, Li et al. [50] used in situ surface-enhanced Raman scattering (SERS) spectroscopy to measure the key reaction intermediates of silver nanoparticles in the oxidation of NO and SO_2_ under the action of NTP (as shown in Figure 6). The DFT calculations are also used to determine the adsorption species, direction, and location.

In order to better understand the interaction mechanisms between plasma and catalyst in an IPC reactor, Wang et al. [51] investigated the discharge processes of a single-bead DBD reactor operating in dry air by optical imaging experiments combined with a numerical fluid model. The results show that magnetic beads with high permittivity can induce local electric field enhancement, which is beneficial to increase the mean electron energy and produce more active species. But it also creates a confined discharge near the contact point of the filled beads, limiting the interaction area between the catalyst and the active plasma species. In addition, the results of the numerical model indicate that the effect of encapsulated beads on charge capture should be taken into account in the equivalent circuit modeling of IPC reactors. These conclusions are important for improving plasma-catalyst synergy.

In 2021, Loenders et al. [52] studied the surface reaction process of different plasma species on Pt catalysts using a micromechanical model, revealing the potential of partial oxidation of CH_4_ to oxides through plasma catalysis. Yi et al. [53] applied a 0D plasma chemical kinetic model to study the main reaction pathway for the oxidation of CH_4_ to methanol in a DBD reactor. In the field of catalytic synthesis of NH_3_, Kedalo et al. [54] studied the efficiency of plasma-assisted vibration-excited heterogeneous nitrogen activation on the Ru surface. A numerical simulation analysis was conducted. The absorption and dissociation energy paths of N_2_ on the Ru catalyst surface were calculated by ab initio molecular dynamics calculations. Furthermore, by solving the chemical kinetic equations of the plasma gas phase and the vibrationally excited molecules on the catalyst surface, the energy cost of the heterogeneous plasma activation of N_2_ is estimated. In order to analyze the role of plasma species in promoting the formation of important intermediates, Sun et al. [55] studied the kinetics of ammonia synthesis using nanosecond pulse discharge plasma combined with catalysts in N_2_/H_2_ mixtures through experiments and a 0D numerical model. A detailed kinetic mechanism consisting of atoms, free radicals, excited species, molecules, ions, and surface species was developed by combining electron-collision reactions, excited-species reactions, ion reactions, direct and dissociative adsorption reactions, and surface reactions. The temporal evolution of species density in N_2_/H_2_ plasma catalytic systems was calculated. However, 0D simulation cannot reflect the impact of the reactor structure on plasma flow and heat transfer in actual processes.

In 2022, Zuo et al. [56] applied a 2D particle/Monte Carlo model to study the propagation mechanism of plasma flow in porous catalysts packed in DBD reactors. The model took into account 19 collision reactions of electrons with O_2_ and N_2_ (including elastic, excitation, ionization, and attachment reactions). The spatial distribution of plasma active species and their evolution over time was analyzed. The influence of the number and size of catalyst pores as well as the catalyst voltage on the plasma discharge process were studied. This research provides a reference method for further understanding the synergistic mechanism between NTP and catalysts. In the field of CO_2_ reforming and utilization, Du et al. [57] established a numerical simulation model of pulsed DBD combined with a catalyst for CO_2_ hydrogenation and analyzed the influence of key intermediates. And their conversion processes on the selective conversion of CH_4_ and CH_3_OH when NTP cooperates with Ni/Cu catalyst were analyzed. Pan et al. [58] established a 0D kinetic model of the IPC reactor, which considered plasma reactions and catalyst surface reactions (adsorption reactions, dissociation reactions, and Langmuir Hinshelwood reactions). The plasma synergistic catalytic mechanism of CH_4_ dry reforming was studied. Based on the numerical model, the transfer of electron energy, active species, and their transformation pathways were analyzed.

**Figure 7:**
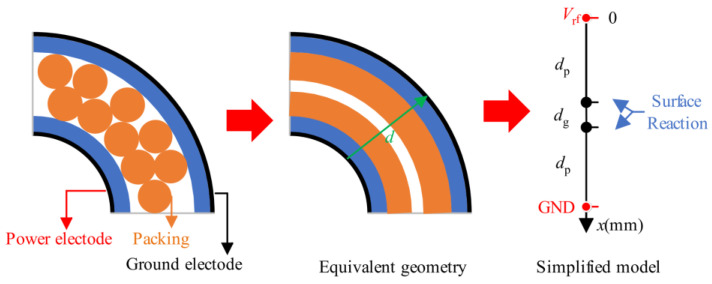
Schematic diagram of a simplified one-dimensional model.

Furthermore, in order to investigate how surface reactions on catalysts affect the distribution of plasma gas-phase species, Zhu et al. [59] extended the 0D numerical model to the 1D plasma fluid numerical model (as shown in Figure 7). The simulation results indicate that catalysts can also affect the spatial distribution of active species, thereby indirectly affecting plasma chemistry. This study solved the problem of the inability of the 0D model to analyze the spatial distribution of active species and achieved an explanation for the redistribution of active species in the NTP synergistic catalyst process. It also provides a reference for the study of plasma catalytic sustainable chemical processes. Khanna et al. [60] established a 2D plasma model using COMSOL to study the graphene growth process in PECVD. The simulation results are of great significance for guiding future experimental research and are conducive to understanding the growth process of graphene and other carbon-based nanostructures in an oxygen-filled plasma environment.

Establishing a numerical simulation model is an important measure to study the synergistic mechanism of NTP and catalysts [61]. In recent years, thanks to the development of detection technology and multi-physical field coupled simulation, the detection and simulation of chemical reaction processes between plasma and catalyst surface have provided conditions for studying the coupling mechanism between NTP and catalyst. However, at present, there is a lack of research on simulation models of the NTP-CH_4_-SCR system. In order to fully understand the synergistic mechanism of NTP and CH_4_-SCR in the synergistic removal process of CH_4_ and NO*_x_*, a more detailed simulation model is still needed.

### 3.3. Research on Energy Consumption Optimization Based on Plasma Products Selectivity

Although NTP technology can improve the low-temperature performance of catalysts, the high energy consumption and unwanted product of NTP technology also need to be noted [62]. At present, for the CH_4_ reduction of NO*_x_* using NTP synergistic catalysts, the energy density of the plasma needs to be in the range of 135~2700 J/L to achieve effective synergistic removal of pollutants [18,19,20,21,22,23,24,25,26,27,28]. For an LNG-diesel dual fuel engine with a power of 5862 kW and an exhaust flow rate of 41,396 kg/h, the energy consumption of the NTP reactor is approximately 21.1% to 421.1% of the engine output power. This indicates that optimizing the energy consumption of the NTP reactor is a necessary measure for introducing NTP to achieve the synergistic removal of CH_4_ and NO*_x_*.

The traditional catalytic reaction is mainly driven by exhaust heat, and the product selectivity of the catalyst is mainly controlled by the active component of the catalyst. For the process of CH_4_ reducing NO*_x_* under the condition of an NTP synergetic catalyst, the operation of an NTP reactor needs additional energy, and its product selectivity is related to the electron energy distribution and electron density. At present, in the process of CH_4_ and NO*_x_* treatment, insufficient attention has been paid to the control of product selectivity of the plasma reaction and the optimization of energy consumption based on this.

**Figure 8:**
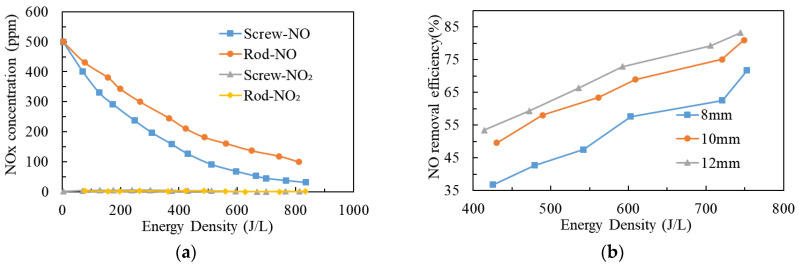
The impact of structural parameters of DBD reactor on NO removal: (**a**) Effect of inner electrode shape on NO removal; (**b**) Effect of inner electrode diameter on NO removal. (Note: Energy density is the ratio of NTP discharge power to gas flow rate).

In the field of desulfurization and denitrification by NTP technology, in order to improve removal efficiency and reduce NTP energy consumption, researchers have conducted relevant research on the structural parameters of NTP reactors. From 2012 to 2015, Wang et al. [63,64,65] used a mixture of NO/N_2_ as simulated exhaust and studied the impact of structural parameters of the DBD reactor on NO removal and energy consumption. The results showed that barrier materials, the diameter of the inner electrode, the shape of the inner electrode, the length of the electrode, and the material of the electrode have significant effects on NO-removal efficiency and energy consumption. Compared to the rod electrode, the screw electrode has a higher electric-field strength at the tooth tips, which can improve NO-removal efficiency (as shown in Figure 8a). And the NTP reactor with an electrode diameter of 12 mm has higher NO-removal efficiency than the NTP reactors with electrode diameters of 8 mm and 10 mm when the same energy density was input (as shown in Figure 8b). Tungsten has a higher secondary electron emission coefficient. Using a tungsten rod as an internal electrode can increase the electron density of NTP, improve NO removal efficiency, and significantly reduce plasma energy consumption. Anaghizi et al. [66,67] also obtained similar results, with the average energy consumption of the screw electrode reduced by 14% compared to the round rod electrode.

**Figure 9:**
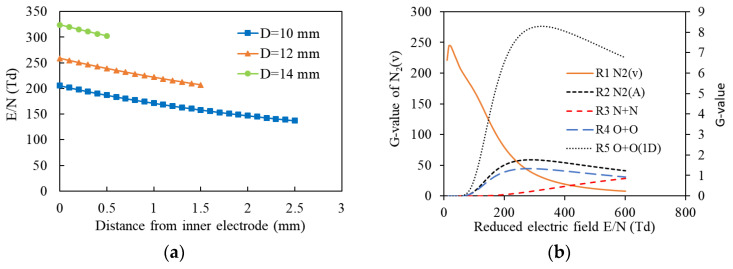
The impact of structural parameters of DBD reactor on E/N and particularly reaction process: (**a**) Effect of inner electrode shape on NO removal; (**b**) The effect of E/N on G-value of the particular reaction process.

From 2018 to 2020, Liu F and Cai YK et al. [68,69] used 860 ppm NO*_x_* (92% NO + 8% NO_2_), 15% O_2_, and N_2_ (balance) to simulate exhaust and studied the impact of structural parameters of NTP reactors on energy consumption and NO oxidation efficiency under oxygen-enriched conditions. The results showed that increasing electrode diameter not only improved the reduced field strength (E/N) and electron mean energy but also made the E/N and electron energy distribution more concentrated (as shown in Figure 9a). This allows the reactor to work at a high O radicals generation efficiency while reducing N_2_(X, v) and N radicals generation (as shown in Figure 9b). Thus, increasing electrode diameter improved NO*_x_* oxidation efficiency, avoided side reactions, and reduced NTP energy consumption. In addition, the electric field strength on the surface of the screwed electrode was much higher than that of the rod electrode, which was conducive to the formation of N radicals. Under oxygen-enriched conditions, N radicals will react with O_2_ to generate NO*_x_*. With the same NO oxidation efficiency, the energy consumption of the screwed electrode is higher.

**Figure 10:**
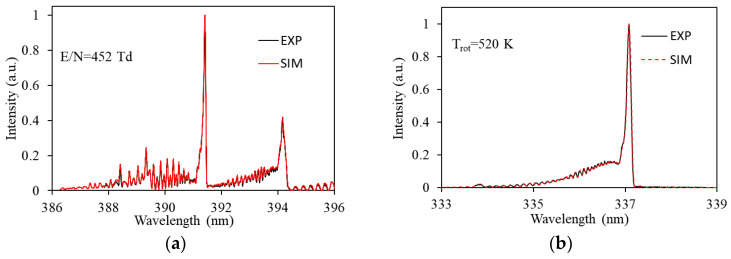
The OES of NTP: (**a**) Spectral measurement of reduced field strength for N_2_^+^(B_2_∑_g_^+^→X_2_∑_g_^+^, 0–0) and N_2_ (C^3^∏_u_→B^3^∏_g_, 2–5); (**b**) N_2_(C^3^∏_u_→B^3^∏_g_, 0–0) Spectroscopic Measurement of Gas Temperature. (Note: EXP is experiment data, SIM is the simulation result).

In 2021, Cai YK et al. [70] used 10% O_2_, 7% CO_2_, 820 ppm NO, 320 ppm SO_2_, 200 ppm C_3_H_6_, 3.1% H_2_O, and N_2_ (balance) to simulate actual diesel exhaust. The OES of N_2_(C^3^∏_u_→B^3^∏_g_, 2−5, 394.3 nm) and N_2_^+^(B_2_∑_g_^+^→X_2_∑_g_^+^, 0−0, 391.4 nm) were measured to detect the E/N in the discharge gap (as shown in Figure 10a). The OES of N_2_(C^3^∏_u_→B^3^∏_g_, 0−0, 337 nm) was measured to detect the gas temperature in the discharge gap (as shown in Figure 10b). The effect and mechanism of inner electrode diameter in the NTP reactor on the oxidation removal of NO and SO_2_ were also studied. The experimental results show that an NTP reactor with larger inner electrodes has a higher E/N and lower gas temperature in the discharge gap, which is conducive to the generation of oxidative radicals (such as O and OH radicals) and the oxidation of NO. Increasing the electrode diameter allowed electrons to obtain more energy while reducing the energy consumption of the NTP reactor.

Previous research has shown that factors such as barrier material, inner electrode material, inner electrode shape, and inner electrode diameter can all affect the energy consumption of NTP reactors for NO*_x_* removal. Improving the yield of target plasma active species and avoiding unnecessary generation of active species can effectively reduce plasma energy consumption while avoiding side reactions. The denitrification mechanism is different, and the required active substances are different. These factors have varying effects on plasma energy consumption and NO*_x_* removal efficiency.

In summary, for the optimization of energy consumption in the integrated removal of CH_4_ and NO*_x_* in an NTP-CH_4_-SCR system, it is necessary to study the mechanism of active species demand in the plasma reaction during the removal process. The structural parameters and discharge parameters of the plasma reactor need to be comprehensively considered to control the product selectivity of the plasma reaction. And enhancing the generation efficiency of effective species can improve the energy utilization efficiency and ultimately realize the optimal control of its energy consumption.

## 4. Conclusions

CH_4_-SCR has been studied as a desirable technology for the removal of NO*_x_* and CH_4_ simultaneously in marine LNG engine exhaust. Compared to MOC+NH_3_-SCR technology, the CH_4_-SCR system has the significant advantages of low cost, a small footprint, and a simple system. The high stability of CH_4_ molecules leads to poor low-temperature activity in the CH_4_-SCR system. The introduction of NTP technology in the CH_4_-SCR system is an effective way to overcome the low exhaust temperature of marine engines and reduce the use of noble metal catalysts. However, the synergistic mechanism between NTP and catalysts is still unclear, which limits the optimization and promotion of energy consumption of the NTP-CH_4_-SCR system. In this paper, the synergistic mechanism of NTP and catalysts in the integrated treatment of CH_4_ and NO*_x_* (including research based on experimental measurements and research based on simulation models) is summarized, and the relevant influencing parameters for energy consumption optimization of NTP reactors are discussed. The following conclusions can be drawn:

(1) The existing research on the synergistic mechanism of NTP and catalysts in the reduction of NO*_x_* by CH_4_ is not fully understood. It is necessary to measure steady-state products before and after the reaction and the intermediate products adsorbed on the catalyst surface. The combination of some catalyst characterization methods (such as TEM, H_2_-TPR, XRD, and XPS), advanced in situ detection techniques (such as DRIFTS, SERS, and OES), and gas component analyzers (such as MS and FTIR) can provide important information, which is of great significance for studying the synergistic mechanism between NTP and catalysts. In addition, these experimental results can provide support for the establishment and verification of numerical models.

(2) In order to fully analyze the interaction mechanism between NTP and catalyst, it is necessary to establish a numerical simulation model. Existing research has shown that 0D, 1D, and 2D simulation models are crucial for studying the interaction between NTP and catalysts. However, in the field of NTP-CH4-SCR, the numerical simulation model is not complete, and the role of highly active unstable products, such as radicals, vibrational states, and excited states in the conversion process of CH_4_ and NO*_x_*, is unclear, which is insufficient to guide the integrated and efficient removal of CH_4_ and NO*_x_*.

(3) The structural parameters of NTP reactors can affect the generation efficiency of active species and energy consumption. And different removal mechanisms require different active species. Existing research has not fully focused on the control of plasma product selectivity and energy consumption optimization in the process of treating CH_4_ and NO*_x_* in the NTP-CH_4_-SCR system.

Therefore, it is necessary to study the synergistic mechanism of NTP combined with a catalyst in CH_4_ and NO*_x_* removal from the exhaust of marine LNG engines and analyze the coupling mechanism between CH_4_ oxidation and NO*_x_* reduction processes in the future. Further, on this basis, it is necessary to refine the demand law and internal mechanism of plasma reaction for efficient removal of CH_4_ and NO*_x_* and complete the optimal control of energy consumption in the plasma catalytic reaction process to improve the removal efficiency of CH4 and NOx at low exhaust temperatures, reduce the use of noble metal catalysts, and achieving low-cost control of greenhouse gases and atmospheric pollutants for marine LNG engines.

## Figures and Tables

**Table 1:** Emissions from high-speed marine LNG engines (manufactured in 2020).

No.	kW–r/min	NO*_x_*(g/kWh)	NMHC * (g/kWh)	CH_4_ (g/kWh)	IMO Tier III for NO*_x_* (g/kWh)
1	120–1500	2.304	0.425	4.427	2.08
2	138–1500	2.085	0.401	4.186	2.08
3	180–1500	2.352	0.508	4.494	2.08
4	210–1500	1.557	0.383	3.224	2.08
5	265–1500	1.703	0.279	2.695	2.08
6	400–1500	2.09	0.531	4.959	2.08

* NMHC is non-methane hydrocarbon emission.

## Data Availability

Not applicable.
